# Anatomy of the distal radioulnar ligament in cats

**DOI:** 10.1177/1098612X221149382

**Published:** 2023-02-13

**Authors:** Rachel M Basa, Paul J Canfield, William R Walsh, Kenneth A Johnson

**Affiliations:** 1 Sydney School of Veterinary Science, Faculty of Science, University of Sydney, NSW, Australia; 2 Surgical and Orthopaedic Research Laboratories, Prince of Wales Clinical School, University of New South Wales, Randwick, NSW, Australia

**Keywords:** Carpus, distal radioulnar ligament, antebrachiocarpal joint, anatomy, supination, pronation

## Abstract

**Objectives:**

The aim of this study was to describe the anatomy of the distal radioulnar ligament in the cat, using gross and histological sections from cadaveric feline carpi.

**Methods:**

Eight feline cadaveric distal radioulnar joints were included in the study, including six that were paraffin- and two that were polymethyl methacrylate-embedded. Each of the sections of the distal radioulnar joint and ligament were viewed macroscopically and microscopically using a dissection microscope and a standard light microscope with polarising capacity.

**Results:**

On gross examination, the distal radioulnar ligament could be seen as a triangular-shaped structure extending between the dorsal surface of the distal radius and ulna. The centre of the ligament had a greater density of tightly packed collagen fibres, while fibrocartilage was identified at the site of both the radial and ulnar entheses. Articular cartilage was noted to extend to the most proximal part of the bulbous portion of the distal ulna and corresponding axial aspect of the distal radius.

**Conclusions and relevance:**

In the cat, there appears to be a less extensive interosseous component of the distal radioulnar ligament compared with the dog and cheetah. Instead, the ligament follows the articular surfaces of the distal radius and ulna. These anatomical differences may account for increased rotation of the feline antebrachium and have clinical implications, particularly with regard to the management of antebrachiocarpal joint injuries.

## Introduction

Cats have greater antebrachial rotation than dogs, and this is thought to be important in activities such as jumping, climbing and grooming.^1^ The presumed causes of this difference include a more flexible interosseous ligament, differences in the collateral ligaments of the elbow and a larger ulnar styloid process.^2,3^ The histology of the distal radioulnar joint and ligament have been investigated in the dog and cheetah, but there have been no similar studies in the domestic cat.^4,5^ In the cheetah, the distal radioulnar ligament is composed of fibrocartilage and the distal radioulnar joint has reduced articular facets that limit rotation of the antebrachium.^5^ In the dog, there is only fibrocartilage in the lateral part of the antebrachiocarpal joint with the ligament itself consisting of a proximal interosseous and distal articular portion.^4^ The aim of this study was to describe the anatomy of the distal radioulnar ligament in the cat using gross and histological sections from cadaveric feline carpi.

## Materials and methods

The carpal joints of eight domestic shorthair cats were collected from the cadavers of cats that were euthanased for reasons unrelated to the study, in accordance with guideline GL001 of the University of Sydney’s animal ethics committee. Inclusion criteria included that the cats were skeletally mature, based on their dentition.

**Figure 1:**
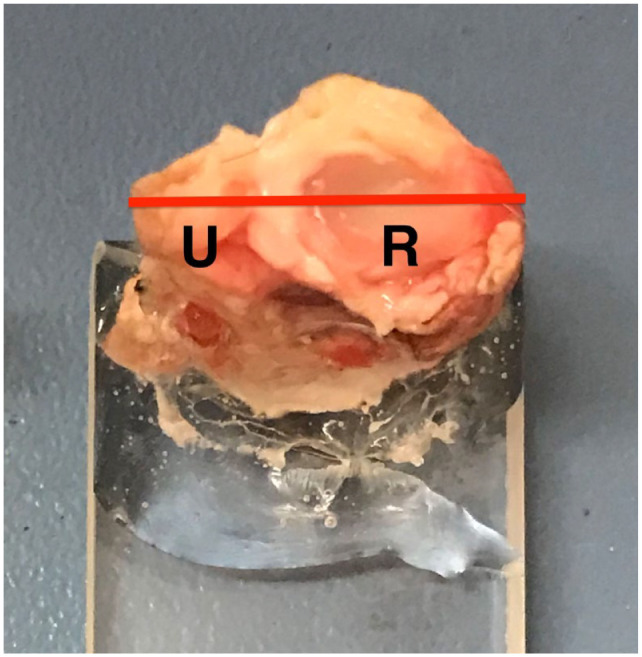
Mounting of a feline distal radioulnar joint on an acrylic block using epoxy resin. The red horizontal line is at the centre of the antebrachiocarpal joint and shows the level at which each specimen was sectioned. The distal radius (R) and ulna (U) were secured to the acrylic block using epoxy resin

For the six limbs that were later paraffin-embedded, the limb was transected through the level of the antebrachiocarpal joint and removed from the remainder of the carpus. Digital images were taken of the gross anatomical specimens. The distal radius and ulna of the specimens, 1 cm proximal to the antebrachoicarpal joint, were fixed to the end of a 5 mm thick acrylic block using epoxy resin (Aradite; Selleys) for 12 h (Figure 1). Once the resin was set, the limbs were sectioned at the level of the distal radio-ulnar joint using an annular diamond blade (Microslice; see Figure 1). Samples were fixed in 10% neutral buffered formalin at room temperature for 72 h and then processed through graded alcohols of 70%, 95% and 100% xylene and infiltrated with paraffin wax. Bones, after fixation, were then decalcified in 10% formic acid/formalin mix at room temperature for 5 days. Specimens were embedded and cut at 4–8 µm onto slides and stained with haematoxylin and eosin, Alcian blue, Masson’s trichome and toluidine blue. The microscope slides were initially viewed using a dissection microscope before being examined with a standard light microscope with polarisable capacity (S261 dissection microscope; Olympus).

**Figure 2:**
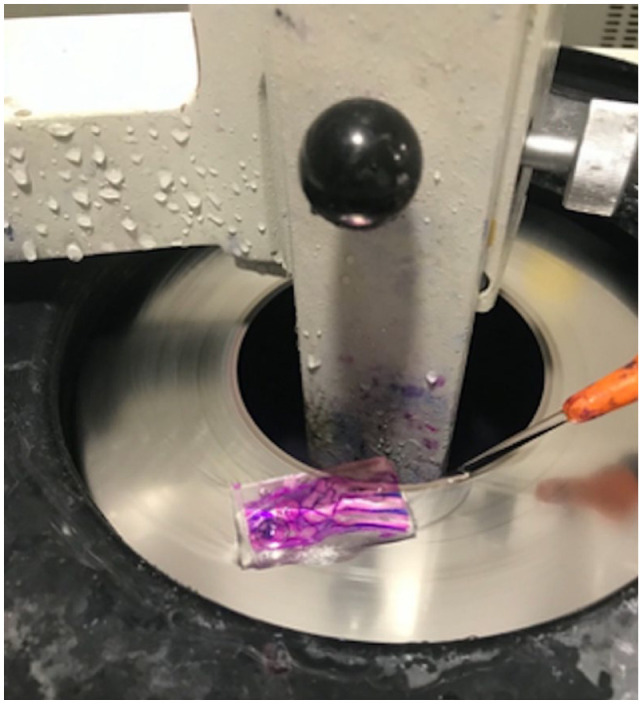
Sectioning of a polymethyl methacrylate specimen using an annular diamond saw blade

For the two limbs that were embedded in polymethyl methacrylate (PMMA), sections were obtained 1 cm proximal to the distal radioulnar joint and 2 cm distal to the distal radioulnar joint. Skin was removed from the specimens; however, joint capsule and tendons were left intact. The sections were fixed in formalin for 3 weeks prior to processing. Following fixation, specimens were dehydrated using increasing concentrations of ethanol that were changed every 2 days (70%, 80%, 90%, 95% and 100%). The samples were infiltrated with methylmethacrylate (Sigma Aldrich) for 10 days and polymerised to effect with PMMA (4 weeks after dehydration). A vacuum chamber was used both before and after the infiltration process, to reduce bubble formation. Embedded specimens were sectioned in the frontal plane of the distal radio-ulnar joint using a Leica SP 1600 microtome (Figure 2). Four sections were collected from each specimen with a thickness of 400 µm. The sections were briefly etched in acidic ethanol (98 ml ethanol 96% and 2 ml HCl 37%) and stained with methylene blue, followed by basic fuchsin. The stained slides were reviewed under low magnification to provide an overview of the section. High magnification was used to assess for the presence of an enthesis and characteristics of the distal radioulnar joint. Polarised light was used to assess the orientation of collagen fibres.

## Results

### Macroscopic description

**Figure 3:**
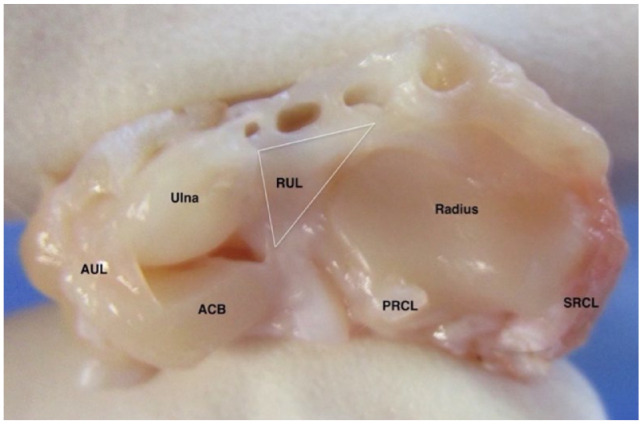
View of the articular surface of the antebrachiocarpal joint, including – in a left to right (lateral to medial) direction – the accessory carpal bone (ACB), ulna and radius. The white triangle shows the basic outline of the distal radioulnar ligament. AUL = accessorioulnar ligament; RUL = radioulnar ligament; PRCL = palmar radiocarpal ligament; SRCL = short radial collateral ligament

The distal radioulnar ligament could be seen as a triangular-shaped structure extending between the dorsal surface of the distal radius and ulna (Figure 3). The proximal extent of the ligament could not be identified due to overlap between the ulnar notch and articular circumference of the radius; however, the distal extent of the interosseous muscle was consistently noted 5 mm proximal to the antebrachiocarpal joint. Palmar to the radioulnar ligament, a ligamentous structure was noted originating from the axial aspect of the ulna styloid process to insert on the base of the accessory carpal bone, consistent with the location of the accessorioulnar ligament. At the articular surface of the antebrachiocarpal joint, the radioulnar ligament fully bridged the space between the distal radius and ulna.

### Microscopic description

**Figure 4:**
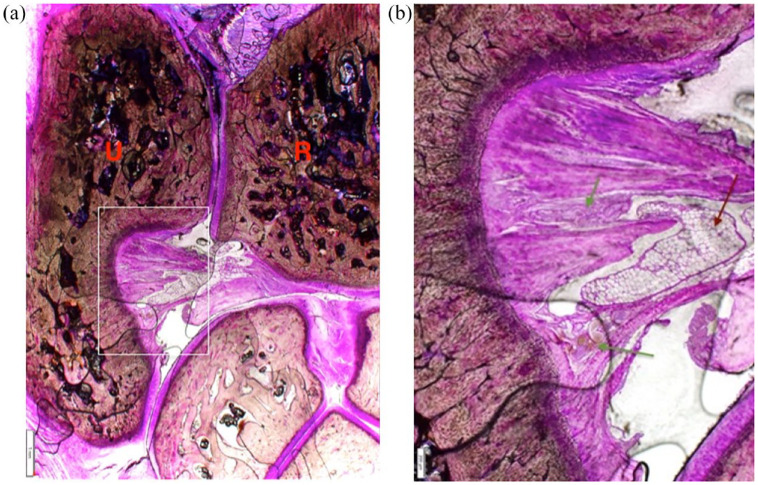
Magnified view of the articular surface of the ulna (U) and part of the distal radioulnar ligament in a polymethyl methacrylate-embedded sample with basic fuchsin stain (400 µm thick). The solid white bar in the bottom left-hand corner of (a) is 1 mm in length; the solid white bar in the bottom left-hand corner of (b) is 200 µm in length. (b) Magnification of the area within the white rectangle in (a), demonstrating the ulna insertion of the distal radioulnar ligament. In (b), the red arrow points to adipose fat, and the green arrows demonstrate the location of two separate clusters of blood vessels, illustrating the are gaps between the collagen fibres of the ligament. R = radius

In the PMMA sections, the radioulnar ligament was identified as collagen fibres with a uniform orientation. In the most dorsal sections, there were spaces parallel to the collagen fibre orientation containing both fat and numerous blood vessels (Figure 4). The palmar sections contained a greater density of collagen fibres.

**Figure 5:**
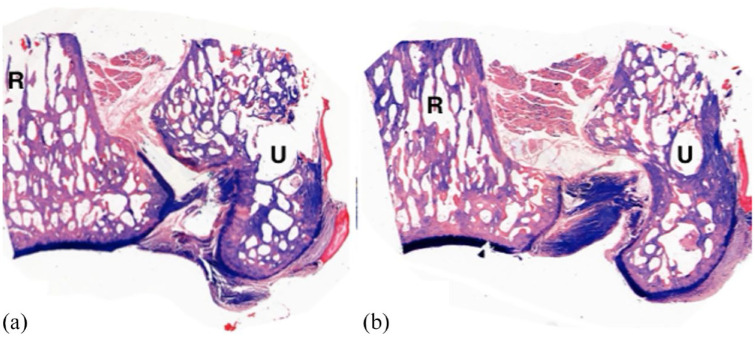
Frontal plane sections of the cat distal radius (R) and ulna (U), low power (× 10 magnification) of two paraffin-embedded sections. (a) A more dorsal section; (b) palmar. The course of the distal radioulnar ligament can be seen as dark purple bands in both (a) and (b), extending between the distal radius and ulna

**Figure 6:**
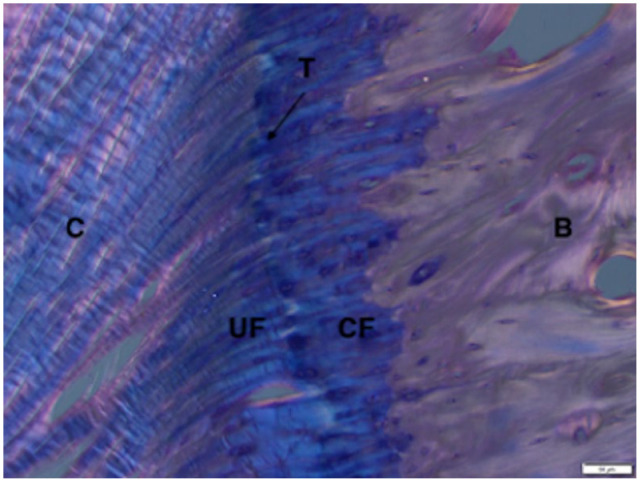
High-power magnification of radial enthesis in a paraffin-embedded section using polarised light and haematoxylin and eosin stain (4 µm slice thickness). Solid white bar at the bottom right-hand corner of the image is 50 µm in length. There are characteristics of a fibrocartilaginous enthesis. C = collagen; UF = uncalcified fibrocartilage; CF = calcified fibrocartilage; T = tidemark (interface line between collagen and uncalcified fibrocartilage); B = bone

**Figure 7:**
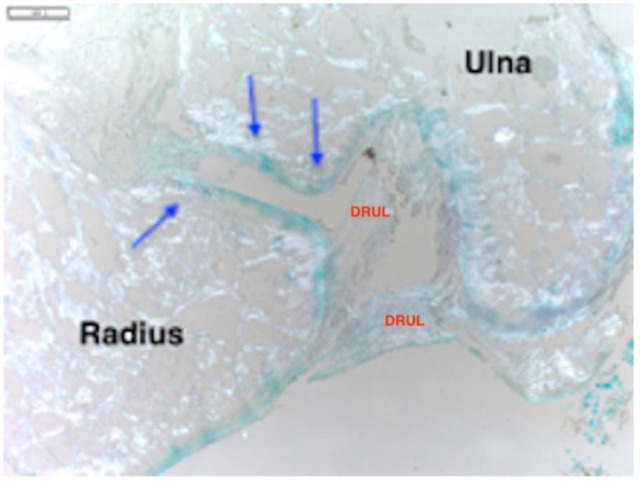
High-magnification image of the feline distal radioulnar joint stained with Alcian blue (4 µm slice thickness from a paraffin-embedded section). The radius and ulna distal articular surfaces are at the bottom of the image. Solid white bar in the top left-hand corner is 1 mm in length. The acid mucins of articular cartilage in this image are stained blue (blue arrows). DRUL = distal radioulnar ligament

In the paraffin-embedded sections, the distal radioulnar ligament extended from the most proximal extent of the articular circumference of the ulna proximally to the level of the antebrachiocarpal joint distally, originating from the radius and inserting on the ulna (Figure 5). The centre of the ligament dorsopalmarally had a greater density of tightly packed collagen fibres vs the edge of the ligament. Fibrocartilage was identified at the site of both the radial and ulnar entheses of the distal radioulnar ligament (Figure 6). There was a wider zone of uncalcified cartilage at the radial insertion and greater density of calcified cartilage at the ulnar insertion of the ligament, both in the frontal plane sections. The extent of the articular surface was assessed using Alcian blue (Figure 7). There was staining uptake to the most proximal extent of the bulbous portion of the distal ulna, and this continued around the abaxial aspect of the distal ulnar articular surface. There was a corresponding articular surface on the distal radius. There was a gradual transition from articular cartilage to periosteum at the proximal extent of the distal radioulnar joint. The interosseous muscle started proximal to the bulbous portion of the distal ulna where the ulnar articular cartilage abruptly ended.

## Discussion

The results of this study confirm that the distal radioulnar joint of the cat is somewhat different than that of the dog, and that there is a true radioulnar ligament rather than a fibrocartilage disc.^4,6^ The microscopic appearance of the feline carpus has previously been described using plastinated specimens; however, the structure of the distal radioulnar joint was not clear.^7^ The radioulnar ligament of the dog has been described as consisting of a proximal interosseous and distal articular portion.^4^ In the cat, there appears to be a less extensive interosseous component; instead, the ligament follows the articular surfaces of the distal radius and ulna.

It has previously been reported that cats have almost double the range of pronation and supination than dogs, which is partly thought to be due to a more flexible and extensive interosseous ligament.^8^ The findings of the present study also seem to support the argument that the composition of the feline distal radioulnar ligament may allow for greater rotation of the distal radius around the ulna. In one experimental study, plate fixation of the radius alone in a simulated pan-carpal arthrodesis did not significantly affect pronation and supination; however, the recommendation of the authors was to avoid burring the distal ulna in order to avoid interfering with antebrachial rotation.^9^ Nevertheless, there are few guidelines regarding the optimal treatment for carpal injuries and the standard of care is largely extrapolated from retrospective or cadaveric studies.^10^

The articular surfaces of the distal radius and ulna are concave, and although the radioulnar ligament provided continuity between the distal radius and ulna, it did not improve congruity at the articular surface, unlike that described in the dog.^6^ In the cat there is also no similar structure to the triangular fibrocartilage, which in people is important in buffering axial carpal forces.^11^ In the cheetah, there is a fibrocartilage disc between the distal radius and ulna, in addition to a shallow ulnar notch that is thought to contribute to reduced supination and pronation.^5^ Unlike a previous description of the dog, there were no fibrocartilage islets identified within the feline radioulnar ligament.^4^

There was a distinct fibrocartilaginous enthesis identified at both the radial and ulnar insertions of the radioulnar ligament. Movement is thought to be the stimulus for metaplasia of fibroblasts to fibrocartilage cells, which would confirm that there is greatest motion at the radial enthesis as confirmed by a wider zone of uncalcified fibrocartilage at the radial insertion.^12^ The greater density of calcified cartilage at the ulnar enthesis suggests that there is greater physiological strength and loading of the ligament at that point.^12^ Although changes in ligament tension during supination and pronation have been investigated in the human wrist, there are currently no studies in the feline carpus to elucidate the implication of these differences in ligament morphology.^13^

While acknowledging the relatively small sample size in this study, it has emphasised the importance of combining thicker PMMA with those of paraffin-embedded specimens in an attempt to overcome their inherent artefacts, namely fragmentation of collagen in paraffin embedding and lack of nuclear detail in PMMA embedding, when studying ligaments and cartilage.

## Conclusions

The feline distal radioulnar ligament has distinct differences from that of the human and dog which may account for differences in rotation of the feline antebrachium. Functional studies are required to further characterise these differences and determine whether there is further impetus to attempt preserving the antebrachiocarpal joint in cases of feline carpal injury.
